# Adaptive Filtering on GPS-Aided MEMS-IMU for Optimal Estimation of Ground Vehicle Trajectory

**DOI:** 10.3390/s19245357

**Published:** 2019-12-05

**Authors:** Haseeb Ahmed, Ihsan Ullah, Uzair Khan, Muhammad Bilal Qureshi, Sajjad Manzoor, Nazeer Muhammad, Muhammad Usman Shahid Khan, Raheel Nawaz

**Affiliations:** 1 Department of Electrical and Computer Engineering, CUI, Abbottabad Campus, Abbottabad 22060, Pakistan; haseeb.ah@gmail.com (H.A.); uzairkhan@cuiatd.edu.pk (U.K.); bilalqureshi@cuiatd.edu.pk (M.B.Q.); 2 Department of Electrical Engineering, MUST, Mirpur 10250 AK, Pakistan; sajjad.ee@must.edu.pk; 3 Department of Mathematics, CUI, Wah Campus, Wah 47000, Pakistan; nazeermuhammad@cuiwah.edu.pk; 4 Department of Computer Science, CUI, Abbottabad Campus, Abbottabad 22060, Pakistan; ushahid@cuiatd.edu.pk; 5 Manchester Interdisciplinary Biocentre, School of Computer Science, Manchester Metropolitan University, Manchester M13 9PR, UK; r.nawaz@mmu.ac.uk

**Keywords:** adaptive kalman filters, global positioning system, inertial navigation system, information fusion, estimation

## Abstract

Fusion of the Global Positioning System (GPS) and Inertial Navigation System (INS) for navigation of ground vehicles is an extensively researched topic for military and civilian applications. Micro-electro-mechanical-systems-based inertial measurement units (MEMS-IMU) are being widely used in numerous commercial applications due to their low cost; however, they are characterized by relatively poor accuracy when compared with more expensive counterparts. With a sudden boom in research and development of autonomous navigation technology for consumer vehicles, the need to enhance estimation accuracy and reliability has become critical, while aiming to deliver a cost-effective solution. Optimal fusion of commercially available, low-cost MEMS-IMU and the GPS may provide one such solution. Different variants of the Kalman filter have been proposed and implemented for integration of the GPS and the INS. This paper proposes a framework for the fusion of adaptive Kalman filters, based on Sage-Husa and strong tracking filtering algorithms, implemented on MEMS-IMU and the GPS for the case of a ground vehicle. The error models of the inertial sensors have also been implemented to achieve reliable and accurate estimations. Simulations have been carried out on actual navigation data from a test vehicle. Measurements were obtained using commercially available GPS receiver and MEMS-IMU. The solution was shown to enhance navigation accuracy when compared to conventional Kalman filter.

## 1. Introduction

The GPS (Global Positioning System) and the INS (Inertial Navigation System) can provide stand-alone navigation solutions; however, both are prone to intrinsic errors/biases which may limit the accuracy and productivity of each navigation technique. The GPS provides fairly precise positioning information but at a low frequency of 1 Hz. It may also suffer from phenomena such as unavailability of a clear line-of-sight to the satellites, multipath propagation/interference in dense urban environments, signal jamming, and degradation in accuracy or complete denial to service by the US government [1,2]. INS, on the other hand, finds preference being a dead reckoning system completely independent of external disturbances, which can provide continuous estimates of not only position and velocity, but also of orientation, at a high measurement output rate. However, it offers only short-term accuracy due to an unbounded growth in error inherent to the inertial sensors. Low cost micro-electro-mechanical-systems-based inertial measurement units (MEMS-IMUs) have extremely poor accuracy and suffer adversely from intrinsic errors such as scale factor, drift, bias, and non-orthogonality. Hence, their outputs contain high noise and large uncertainties. As a result, errors inherent to a low-cost IMU in terms of attitude, velocity, and position, grow swiftly in stand-alone mode [3]. Moreover, being a dead reckoning system, the INS requires accurate initialization and alignment [4,5], which in itself, presents a challenge without an external aid, if low-cost MEMS-based IMUs are used [6,7].

Precise estimation remains an essential requirement for any navigation system, especially applications related to autonomous navigation [8,9,10] and the smart city concept [11,12]. Calibrating an inertial sensor can considerably improve its accuracy as the majority of the uncertainties are associated with the error behavior of a sensor. However, one of the most commonly used methodologies to improve accuracy is augmenting the INS with some aiding source; for example, the GPS [13,14,15] Dead-reckoning using data-fusion on GPS-aided IMU can be used for greater accuracy, reliability, and robustness against jamming [16]. For development of smaller, reliable, and low-cost navigation systems, while also targeting global competitiveness in consumer market, it is inevitable to utilize inexpensive GPS receivers and inertial sensors, especially low grade MEMS-based IMUs, even though they provide less accurate motion information.

This paper proposes a novel framework based on adaptive Kalman filtering, implemented using the GPS-INS loose-coupling integration strategy due to its simplicity, robustness, and comparatively lesser computational load. System modeling has been carried out by implementation of position, velocity, and attitude error models of the inertial sensors to enhance estimation accuracy. The proposed scheme is based on a hybrid of the Sage-Husa filter and the strong tracking filter. The proposed algorithm offers a balance between accuracy and strong convergence to optimum estimation for highly dynamic systems. It is a practical algorithm and provides a reliable, accurate, and cost-effective solution, as shown by simulations on real vehicle navigation data. The strategy was verified practically by aiding a low-cost MEMS-IMU (YH-7000 commercial grade MEMS IMU) with Novatel’s OEMV Series GPS receiver.

The paper is organized as follows: Section 2 provides a brief account of GPS-INS loose coupling scheme, conventional linear Kalman filtering, and adaptive Kalman filtering. Algorithms for Sage-Husa and strong tracking filters are also explained. Afterwards, GPS and INS models are discussed. The proposed scheme is explained in Section 3, along with system model by implementing IMU position; velocity and attitude errors; an earth model; and gravity correction. Simulations and results are presented in Section 4, while the discussion is concluded in Section 5.

## 2. Inertial Navigation Mechanism

Inertial navigation requires determining a vehicle’s attitude and position in a three-dimensional frame of reference. This is accomplished by using three perpendicularly mounted gyroscopes to provide rate-of-turn in three different axes, while three orthogonally mounted accelerometers provide accelerations in these three axes as experienced by the vehicle. Measurements provided by the gyroscopes are with respect to the inertial frame of reference. However, accelerometers cannot provide the acceleration with respect to the inertial frame only. Their measurements also include a component of acceleration caused by the gravitational force. Measurement provided by an accelerometer can be written as difference between the true acceleration of a body in space and the acceleration due to gravitational pull; that is,

(1) f=a−g.

This quantity is known as the specific force, and can be described as the non-gravitational force exerted on per unit mass of the body. The equation can be rearranged to calculate the true acceleration. This equation is termed the navigation equation in literature (and can take different forms depending upon the choice of reference frame). If r is the unit vector representing the vehicle’s position from origin, then acceleration of the vehicle in an inertial frame is given by:

(2) $$\frac{d^{2}r}{dt^{2}}=f_{i}+g_{i}.$$

However, when the navigation process is being carried out in a non-fixed rotating frame of reference, such as the Earth frame, additional forces act on the vehicle as a function of the rotation of the reference frame. This effect is known as the Coriolis effect, and the velocity of vehicle can then be calculated using the inertial velocity as follows:

(3) $$v_{e}=\frac{dr}{dt}=v_{i}-\omega_{ie}\times r,$$

where $\omega_{ie}={\left[\;0\;0\;\Omega_{e}\;\right]}^{T}$ represents the Earth’s rate of turn with respect to the inertial frame, and the symbol × indicates vector product. The symbol $\Omega_{e}=15.041067$°/hr is the Earth’s angular speed. The specific force measurements provided by accelerometers are expressed in the vehicle’s body frame as:

(4) $$f_{b}={\left[\;f_{x}\;f_{y}\;f_{z}\;\right]}^{T}.$$

Specific force measurements from body frame represented by the subscript, *b*, are then transformed to the local geodetic or the navigation frame using the transformation matrix, $C_{b}^{n}$:

(5) $$f_{n}=\left[\begin{array}{c} f_{N}\\ f_{E}\\ f_{D} \end{array}\right]=C_{b}^{n}f_{b},$$

where the subscript *b* and superscript *n* of the transformation matrix, $C_{b}^{n}$, denote body and frame references, respectively, and the transformation matrix is given by,

(6) $$C_{n}^{b}=C_{b}^{nT}=\;\begin{array}{cc} \; & \left[\begin{array}{cc} \cos\psi \cos\varphi -\sin\psi \sin\theta \sin\varphi & -\sin\psi \cos\theta \\ \sin\psi \cos\varphi +\cos\psi \sin\theta \sin\varphi & \cos\psi \cos\theta \\ -\cos\theta \sin\varphi & \sin\theta \end{array}\right.\\ \; & \;\;\left.\begin{array}{c} \cos\psi \sin\varphi +\sin\psi \sin\theta \cos\varphi \\ \sin\psi \sin\varphi -\cos\psi \sin\theta \cos\varphi \\ \cos\theta \cos\varphi \end{array}\right], \end{array}$$

where φ, θ, and ψ are vehicle’s roll, pitch, and yaw angles, respectively. However, the three acceleration components expressed in the navigation frame cannot directly provide velocity components in the navigation frame. This is due to three reasons: (1) The Earth’s rotation rate, which is expressed as angular velocity vector $\omega_{ie}^{n}$ in the local navigation frame. (2) There is a change in orientation of the local navigation frame with respect to the Earth’s frame, which is because of the definition of the local level north and vertical directions. The local north is at a tangent to the Earth’s meridian at all times, whereas the vertical direction is perpendicular to the Earth’s surface. This effect can be expressed as the angular velocity vector $\omega_{en}^{n}$, which is the turn-rate of the navigation frame with respect to the Earth-fixed frame, and is known as the transport rate. (3) the third reason is gravity of the Earth, expressed in terms of the local level gravity vector, $g_{l}^{n}$. Mathematically, these three factors can be expressed as

(7) $$\omega_{ie}^{n}=\left[\begin{array}{c} \Omega_{e}\cos L\\ 0\\ \Omega_{e}\sin L \end{array}\right],$$

where *L* represents vehicle’s latitude.

(8) $$\omega_{en}^{n}=\left[\begin{array}{c} \frac{v_{E}}{R+h}\\ -\frac{v_{N}}{R+h}\\ -\frac{v_{E}\tan L}{R+h} \end{array}\right],$$

where $v_{N}$ and $v_{E}$ represent vehicle’s north and east velocity components, respectively; R=6,378,137 m is the equatorial radius of Earth; and h is the height of vehicle above the Earth’s surface.

(9) $$g_{l}^{n}=\left[\begin{array}{c} 0\\ 0\\ g \end{array}\right],$$

where *g* is obtained from the normal gravity model, which is a well-known expression [16], given as:

(10) $$g=a_{1}\left(1+a_{2}\sin^{2}\varphi +a_{3}\sin^{4}\varphi \right)+\left(a_{4}+a_{5}\sin^{2}\varphi \right)h+a_{6}h^{2},$$

where the coefficients $a_{1}$ through $a_{6}$ are constants given as; $a_{1}$ = 9.7803267714 m/s${}^{2}$, $a_{2}=0.0052790414$, $a_{3}$ = 0.0000232718, $a_{4}$ = −0.0000030876910891/s${}^{2}$, $a_{5}=0.000000004397731$/s${}^{2}$, and $a_{6}=0.000000000000721$/ms${}^{2}$.

Taking into account all the factors discussed above, the rate of change of vehicle’s north, east and down (NED) velocities in a navigation frame can be described as:

(11) $${\dot{v}}_{N}=f_{N}-2\Omega_{e}v_{E}\sin L+\frac{v_{N}v_{D}-v_{E}^{2}\tan L}{R+h}+g$$

(12) $${\dot{v}}_{E}=f_{E}+2\Omega_{e}\left(v_{N}\sin L+v_{D}\cos L\right)+\frac{v_{E}\left(v_{D}+v_{N}\tan L\right)}{R+h}-g$$

(13) $${\dot{v}}_{D}=f_{D}-2v_{E}\cos L+\frac{v_{E}^{2}+v_{N}^{2}}{R+h}+g.$$

The position of the navigating vehicle can be expressed in terms of latitude $\left(\dot{L}\right)$, longitude $\left(\dot{l}\right)$, and height $\left(\dot{h}\right)$ above the Earth’s surface as:

(14) $$\dot{L}=\frac{v_{N}}{R+h}$$

(15) $$\dot{l}=\frac{v_{E}\sec L}{R+h}$$

(16) $$\dot{h}=-v_{D}.$$

**Figure 1:**
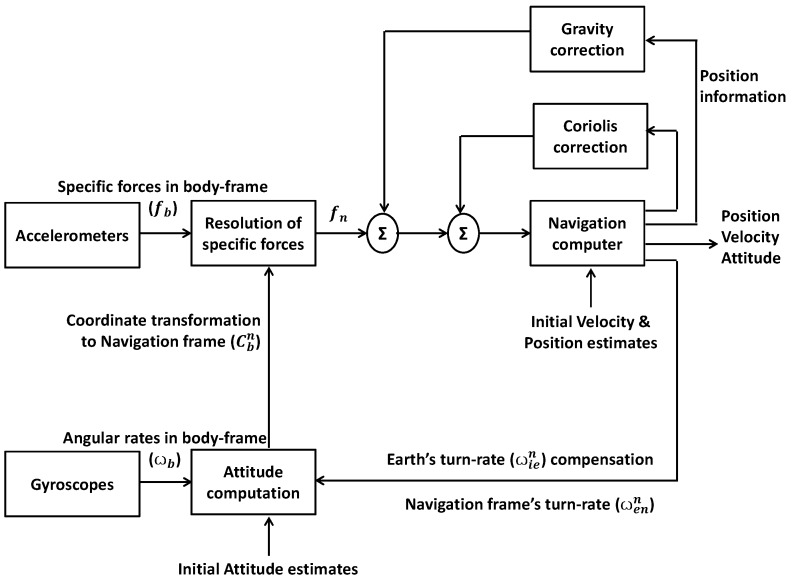
Inertial navigation mechanism.

The whole principle of strap-down inertial navigation in the local navigation frame is demonstrated by the schematic diagram in Figure 1. The GPS model considered in this research can be found in [17].

## 3. Filtering

Development of a reliable aided inertial navigation system depends on selecting an appropriate estimation method. The quality of parameter estimates depends chiefly on the type and accuracy of inertial sensors. Thus, the information from sensors should adequately be formulated with careful consideration of error characteristics in each sensor. Based upon the type of coupling, there are two basic GPS/INS integration modes; namely, loose and tight coupling modes [18]. The loosely coupled integration is the simplest and most robust technique of coupling. Navigation solutions are generated by both the INS and the GPS receiver, independently. A third navigation solution is then derived by using an estimator to merge the information provided by the two independent solutions. The Kalman filter including its derivatives is the most widely used filter to realize this fusion [19,20,21]. The fusion Kalman filter calculates error estimates between the two solutions, which are fed back to provide corrected attitude, velocity and position. The robustness of the loosely coupled scheme is due to the fact that if one system fails, the other sensor can still provide navigation, although with reduced accuracy. This research work utilizes the loose coupling strategy for INS-GPS fusion due to its simplicity, robustness, and lesser computational load.

### 3.1. Conventional Kalman Filter

The Kalman filter is a mathematical estimator used for predicting a system’s behavior from noisy observations. It is a recursive algorithm which uses a series of steps, involving prediction and updating, to obtain an optimal minimum variance estimate of a system or state vector. It is commonly used for estimation in the field of navigation, particularly for autonomous applications [22,23]. It combines noisy sensor outputs (such as those from GPS, accelerometers, and gyroscopes measurements) and estimates the dynamic state of a system (which may include a vehicle’s attitude, acceleration, velocity, position, etc.). For a discrete-time system, the Kalman filter algorithm is given by the following expressions:

Prediction step:

(17) $${\hat{x}}_{k+1|k}=\Phi_{k+1|k}{\hat{x}}_{k|k}$$

(18) $$P_{k+1|k}=\Phi_{k+1|k}P_{k|k}\Phi_{k+1|k}^{T}+Q_{k}$$

Measurement update step:

(19) $$K_{k+1}=P_{k+1|k}H_{k+1}^{T}{\left[H_{k+1}P_{k+1|k}H_{k+1}^{T}+R_{k+1}\right]}^{-1}$$

(20) $$P_{k+1|k+1}=\left[I-K_{k+1}H_{k+1}\right]P_{k+1|k}$$

(21) $${\hat{x}}_{k+1|k+1}={\hat{x}}_{k+1|k}+K_{k+1}\left[z_{k+1}-H_{k+1}{\hat{x}}_{k+1|k}\right],$$

where Φ is the n×n state transition matrix; ${\hat{x}}_{k|k}$ and ${\hat{x}}_{k+1|k}$ represent the corrected(or *a posteriori*) value and the predicted (or *a priori*) value of the estimated state vector *x*, respectively; $P_{k+1|k+1}$ and $P_{k+1|k}$ are the *a posteriori* and *a priori* state covariance matrices of order n×n, respectively, at time $t_{k}$; and the matrix $K_{k+1}$ of order n×m is the Kalman gain. The term $\left[z_{k+1}-H_{k+1}{\hat{x}}_{k+1|k}\right]$ is called the residual or measurement innovation, and $H_{k}$ is the measurements matrix of order m×n. The matrices *Q* (of order n×n) and *R* (of order m×m) represent the system and measurement noise covariances, respectively. Assuming that system noise ($w_{k}$) and measurement noise ($v_{k}$) are uncorrelated and have characteristics of white noise, we can write the mean matrices as:

(22) $$E[w_{k}]=0$$

(23) $$E[v_{k}]=0.$$

And covariance matrices can be written as:

(24) $$Cov\left[w_{k},w_{j}\right]=\left\{\begin{array}{c} Q_{k},j=k\\ 0,j\neq k \end{array}\right.$$

(25) $$Cov\left[v_{k},v_{j}\right]=\left\{\begin{array}{c} R_{k},j=k\\ 0,j\neq k \end{array}\right.$$

(26) $$Cov[w_{k},v_{j}]=0,\forall j,k.$$

### 3.2. Adaptive Kalman Filtering

Due to its reliance on a priori information (of dynamic model and noise parameters), the Kalman filter may suffer from degradation or even divergence in situations where this *a priori* information is time-varying (or unknown) [24]. The matrix *R* usually contains information provided in a sensor’s data sheet, while the matrix *Q* is attained analytically. Thus, any errors in *Q* and/or *R* may lead to sub-optimal estimation. Although usually taken as constants, *Q* and *R* for most practical situations must be adjusted to incorporate time-varying states and sensor inaccuracies which also vary under different dynamic conditions [25,26]. Similarly, in situations where system states are changing abruptly, robustness of the filter also becomes very critical, so that the filter can keep up with sharp changes in observations/measurements. This is achieved by adjusting Kalman gain *K*, or process covariance matrix P [27]. In order to achieve strong convergence, filter accuracy may have to be suppressed in such cases. This type of filtering is known as adaptive Kalman filtering

#### 3.2.1. Sage-Husa Adaptive Kalman Filter

The Sage-Husa Adaptive Kalman filter [28] is based on a maximum *a priori* estimation technique. Since the old measurements can influence the estimation process, the filter performance can be optimized by utilizing a finite set of recent measurements. This is achieved by assigning weights to *Q* and *R* matrices, which causes the filter to adjust the gain K adaptively. So, in situations where noise statistics vary with time, increasing the weights of latest innovations will lead to enhanced estimation accuracy. For systems with time varying noise characteristics, we can write the mean matrices as

(27) $$E\left[w_{k}\right]=q_{k}$$

(28) $$E\left[v_{k}\right]=r_{k}.$$

Filtering functions for Sage-Husa–Kalman filter are:

Prediction step:

(29) $${\hat{x}}_{k+1|k}=\Phi_{k+1|k}{\hat{x}}_{k|k}+{\hat{q}}_{k+1}$$

(30) $$P_{k+1|k}=\Phi_{k+1|k}P_{k|k}\Phi_{k+1|k}^{T}+Q_{k}.$$

Measurement update step:

(31) $$K_{k+1}=P_{k+1|k}H_{k+1}^{T}{\left[H_{k+1}P_{k+1|k}H_{k+1}^{T}+R_{k+1}\right]}^{-1}$$

(32) $$P_{k+1|k+1}=\left[I-K_{k+1}H_{k+1}\right]P_{k+1|k}$$

(33) $${\tilde{z}}_{k}=z_{k+1}-H_{k+1}{\hat{x}}_{k+1|k}-{\hat{r}}_{k+1}$$

(34) $${\hat{x}}_{k+1|k+1}={\hat{x}}_{k+1|k}+K_{k+1}{\tilde{z}}_{k},$$

where

(35) $${\hat{r}}_{k+1}=\left(1-d_{k}\right){\hat{r}}_{k}+d_{k}\left(z_{k+1}-H_{k+1}{\hat{x}}_{k+1|k}\right)$$

(36) $${\hat{R}}_{k+1}=\left(1-d_{k}\right){\hat{R}}_{k}+d_{k}({\tilde{z}}_{k+1}{\tilde{z}}_{k+1}^{T}-H_{k+1}P_{k+1|k}H_{k+1}^{T}$$

(37) $${\hat{q}}_{k+1}=\left(1-d_{k}\right){\hat{q}}_{k}+d_{k}({\hat{x}}_{k+1}-\Phi_{k+1|k}{\hat{x}}_{k}$$

(38) $$\begin{array}{ccc} {\hat{Q}}_{k+1} & = & \left(1-d_{k}\right){\hat{Q}}_{k}+d_{k}(K_{k+1}{\tilde{z}}_{k+1}{\tilde{z}}_{k+1}^{T}K_{k+1}^{T}\\ & & +P_{k+1}-\Phi_{k+1|k}P_{k|k}\Phi_{k+1|k}^{T}). \end{array}$$

The parameter *d* is known as the attenuation factor, given by

(39) $$d_{k}=\frac{1-b}{1-b^{k+1}},0<b<1,$$

where *b* is called the forgetting factor. Generally, *b* is selected using trial and error method but a value between 0.95 and 0.99 has been found to work well [29] to converge the filter to optimal *Q* and *R*.

#### 3.2.2. Strong Tracking Robust Kalman Filter

When dealing with inaccurate linear or non-linear dynamic system models, or in situations where state changes are more sudden, the strong tracking filter may provide another effective method for adaptive state estimation [27]. Although lacking in extensive theoretical analysis, the filter has widely been used in practical applications. It makes use of a time varying fading factor which is computed from the most recent measurement innovation, and dynamically corrects the error covariance for state prediction, providing strong tracking of a maneuvering target with abrupt state changes.

The strong tracking Kalman filter algorithm updates the system covariance matrix *P* by suppressing the filter accuracy, but making it more robust for real time situations, as generalized below:

(40) $$P_{k+1|k}=\lambda_{k+1}\Phi_{k+1|k}P_{k}\Phi_{k+1|k}^{T}+Q_{k},$$

where the parameter λ is called the fading factor and is calculated upon the arrival of new measurements, as proposed in [30].

(41) $$\lambda_{k+1}=diag\left[\lambda_{1(k+1)},\lambda_{2(k+1)},\lambda_{3(k+1)},\ldots \lambda_{n(k+1)}\right],$$

where

(42) $$\lambda_{i(k+1)}=\left\{\begin{array}{c} C_{k+1},if\;C_{k+1}>1\\ 1,if\;C_{k+1}\leq 1, \end{array}\right.$$

and $C_{k+1}$ is defined as

(43) $$C_{k+1}=\frac{Tr[v_{o(k+1)}-R_{k+1}-H_{k+1}Q_{k}H_{k+1}^{T}]}{Tr[\Phi_{k+1|k}P_{k}\Phi_{k+1|k}^{T}H_{k+1|k}^{T}H_{k+1}]},$$

and $v_{o(k+1)}$ is the measurement innovation given by

(44) $$v_{o(k+1)}=\left\{\begin{array}{c} {\tilde{z}}_{1}{\tilde{z}}_{1}^{T},\left(k=0\right)\\ \frac{\rho v_{o(k)}+{\tilde{z}}_{k+1}{\tilde{z}}_{k+1}^{T}}{1+\rho },\left(k\geq 1,0\leq \rho <1\right). \end{array}\right.$$

The value of parameter ρ is generally taken to be 0.95–0.99. The filter is active only when the system is experiencing abrupt state change (λ>1); that is, when

(45) $$\begin{array}{c} Tr[v_{o(k+1)}-R_{k+1}-H_{k+1}Q_{k}H_{k+1}^{T}]>\\ Tr[\Phi_{k+1|k}P_{k}\Phi_{k+1|k}^{T}H_{k+1|k}^{T}H_{k+1}]. \end{array}$$

## 4. Proposed Scheme

**Figure 2:**
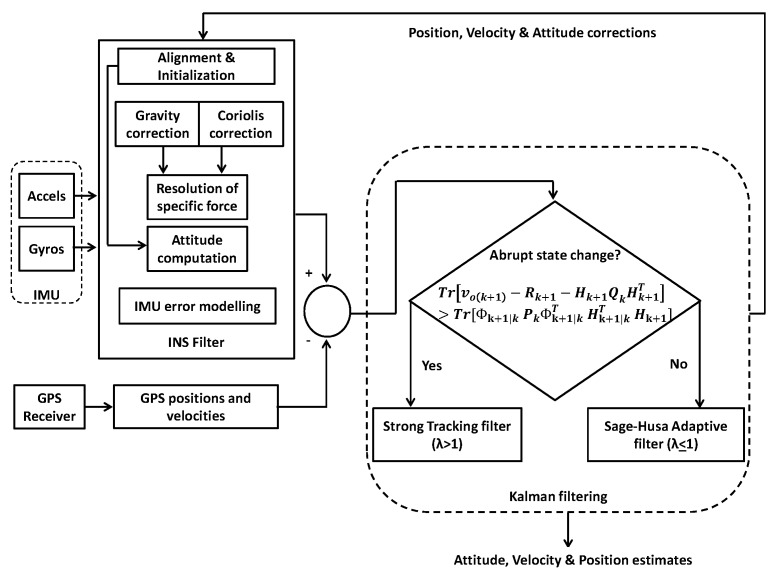
Proposed scheme.

A hybrid filter based on Sage-Husa and strong tracking Kalman filters is presented which offers balance between accuracy and robustness in practical real-time situations where variation in noise statistics is also accompanied by sudden changes in system states. When state change is not abrupt (λ≤1), the filter will prioritize estimation by Sage-Husa algorithm; otherwise, when λ>1, estimation will be carried out through strong tracking filter. The flow diagram demonstrates the filter operation. The filter has been implemented using a loosely coupled scheme due to its simplicity and robustness. The proposed algorithm is depicted in Figure 2.

### Mathematical Model

In state-space representation for a linear dynamic system, a system of n-order differential equations is transformed into a set of n-coupled, first-order differential equations. Similarly, for the case of INS-GPS integration, the differential equations representing INS errors can be expressed in a state-space form, which will then be used as system model for Kalman filter. The state-space model consists of two equations; i.e., a dynamic equation and a measurement equation, which can be written respectively as:

(46) $$\dot{x}=Fx+w$$

(47) z=Hx+v,

where *x* is state vector of order n×1, *F* is called system dynamic matrix with order n×n; *w*, an n×1 vector, represents the system noise; *z* and *v* are called the observation vector and the measurement noise vector, respectively, both of order m×1. *H* is the measurement matrix of order m×n, with *m* measurements and *n* states. The above equations can be written in discrete form as:

(48) $$x_{k+1}=\Phi_{k+1}x_{k}+w_{k}$$

(49) $$z_{k}=H_{k}x_{k}+v_{k},$$

where $x_{k}$ is the state vector of order n×1 at time $t_{k}$; $\Phi_{k+1}$ is the n×n state transition matrix at time $t_{k+1}$; $w_{k}$ is the system noise of order n×1; $z_{k}$ is the observation vector of order m×1; $H_{k}$ is the measurements matrix of order m×n; and $v_{k}$ is the m×1 measurement noise vector at time $t_{k}$. For a discrete-time system, the state transition matrix can be written as:

(50) $$\Phi =e^{F△t}.$$

Taylor series expansion of this equation over a small time interval △t yields the following expression (ignoring the higher terms):

(51) Φ=I+F△t,

where *I* is the identity matrix. The system matrix *F* is a 9×9 matrix formed by incorporating the attitude, position and velocity error models of the inertial sensors, which are explained by Titterton in [31]. The system matrix *F* is given by

(52) $$F=\left[\begin{array}{ccc} F_{1} & F_{2} & F_{3}\\ F_{4} & F_{5} & F_{6}\\ F_{7} & F_{8} & F_{9} \end{array}\right],$$

where $F_{1}$ through $F_{9}$ are all 3×3 matrices given as

(53) $$F_{1}=\left[\begin{array}{ccc} 0 & -(\Omega S_{L}+\frac{v_{E}T_{L}}{r_{E}} & \frac{v_{N}}{r_{N}}\\ \Omega S_{L}+\frac{v_{E}T_{L}}{r_{E}} & 0 & \Omega C_{L}+\frac{v_{E}}{r_{E}}\\ -\frac{v_{N}}{r_{N}} & -(\Omega C_{L}+\frac{v_{E}}{r_{E}}) & 0 \end{array}\right]$$

(54) $$F_{2}=\left[\begin{array}{ccc} 0 & \frac{1}{r_{N}} & 0\\ -\frac{1}{r_{N}} & 0 & 0\\ 0 & -\frac{T_{L}}{r_{E}} & 0 \end{array}\right]$$

(55) $$F_{3}=\left[\begin{array}{ccc} -\Omega S_{L} & 0 & -\frac{v_{E}}{r_{E}^{2}}\\ 0 & 0 & \frac{v_{N}}{r_{N}^{2}}\\ -(\Omega C_{L}+\frac{v_{E}}{r_{E}C_{L}^{2}}) & 0 & \frac{v_{E}T_{L}}{r_{E}^{2}} \end{array}\right]$$

(56) $$F_{4}=\left[\begin{array}{ccc} 0 & -f_{D} & f_{E}\\ f_{D} & 0 & -f_{N}\\ -f_{E} & f_{N} & 0 \end{array}\right]$$

(57) $$F_{5}=\left[\begin{array}{ccc} \frac{v_{D}}{r_{N}} & -2(\Omega S_{L}+\frac{v_{E}T_{L}}{r_{E}}) & \frac{v_{N}}{r_{N}}\\ 2\Omega S_{L}+\frac{v_{E}T_{L}}{r_{E}} & \frac{v_{N}T_{L}+v_{D}}{r_{N}} & 2\Omega C_{L}+\frac{v_{E}}{r_{E}}\\ -\frac{2v_{N}}{r_{N}} & -2(\Omega C_{L}+\frac{v_{E}}{r_{E}}) & 0 \end{array}\right]$$

(58) $$F_{6}=\left[\begin{array}{ccc} -v_{E}\left(2\Omega C_{L}+\frac{v_{E}}{r_{E}C_{L}^{2}}\right) & 0 & \frac{v_{E}^{2}T_{L}}{r_{E}^{2}}-\frac{v_{N}v_{D}}{r_{N}^{2}}\\ 2\Omega \left(v_{N}C_{L}-v_{D}S_{L}\right)+\frac{v_{N}v_{E}}{r_{E}C_{L}^{2}} & 0 & -\frac{v_{E}\left(v_{N}T_{L}+v_{D}\right)}{r_{E}^{2}}\\ 2\Omega v_{E}S_{L} & 0 & \frac{v_{N}^{2}+v_{E}^{2}}{r_{E}^{2}} \end{array}\right]$$

(59) $$F_{7}=0_{3x3}$$

(60) $$F_{8}=\left[\begin{array}{ccc} \frac{1}{r_{N}} & 0 & 0\\ 0 & \frac{1}{r_{E}C_{L}} & 0\\ 0 & 0 & -1 \end{array}\right]$$

(61) $$F_{9}=\left[\begin{array}{ccc} 0 & 0 & -\frac{v_{N}}{r_{N}^{2}}\\ \frac{v_{E}T_{L}}{r_{E}C_{L}} & 0 & -\frac{v_{E}}{r_{E}^{2}C_{L}}\\ 0 & 0 & 0 \end{array}\right],$$

where $f_{N}$, $f_{E}$, and $f_{D}$ are the north, east, and down components of specific force, whereas, $v_{N}$, $v_{E}$, and $v_{D}$ are the north, east, and down components of vehicle’s velocity, respectively. Ω=15.041067${}^{\circ }$/hr represents the Earth’s angular speed. $r_{N}=R_{N}+h$ and $r_{E}=R_{E}+h$, where $R_{N}$ and $R_{E}$ are known as Earth’s meridian radius of curvature and transverse radius of curvature, respectively, when an ellipsoidal model is considered for Earth. $S_{L}$, $C_{L}$, and $T_{L}$ represent sin, cos, and tan of the vehicle’s latitude (*L*), respectively.

(62) $$R_{N}=\frac{R_{e}\left(1-e^{2}\right)}{{\left(1-e^{2}\sin^{2}L\right)}^{\left(3/2\right)}}$$

(63) $$R_{E}=\frac{R_{e}}{{\left(1-e^{2}\sin^{2}L\right)}^{\left(1/2\right)}},$$

where *e* is the eccentricity of the Earth, with a constant value of e=0.08181919082423, and $R_{e}$ is known as the equatorial radius of the earth, with value $R_{e}=6378137$ m. The state vector *x* is given by

(64) $$x={\left[\begin{array}{ccccccccc} \delta \alpha & \delta \beta & \delta \gamma & \delta v_{N} & \delta v_{E} & \delta v_{D} & \delta L & \delta l & \delta h \end{array}\right]}^{T},$$

where δα, δβ, and δγ are the errors in roll, pitch, and yaw; $\delta v_{N}$, $\delta v_{E}$, and $\delta v_{D}$ are the errors in north, east, and down velocities; and δL, δl, and δh are the errors in latitude, longitude, and altitude, respectively.

The system noise matrix *Q* consists of variance errors in its principal diagonal and is formed directly from the IMU sensor data sheet, whereas the uncertainties in the GPS measurements are used to derive the measurement noise covariance matrix *R*.

(65) $$Q=diag\left[\begin{array}{ccccccccc} \sigma_{w_{x}}^{2} & \sigma_{w_{y}}^{2} & \sigma_{w_{z}}^{2} & \sigma_{a_{x}}^{2} & \sigma_{a_{y}}^{2} & \sigma_{a_{z}}^{2} & \sigma_{a_{x}}^{2} & \sigma_{a_{y}}^{2} & \sigma_{a_{z}}^{2} \end{array}\right]$$

(66) $$R=diag\left[\begin{array}{cccccc} \sigma_{v_{N}}^{2} & \sigma_{v_{E}}^{2} & \sigma_{v_{D}}^{2} & \sigma_{L}^{2} & \sigma_{l}^{2} & \sigma_{h}^{2} \end{array}\right].$$

The difference between GPS and IMU velocity and position measurements forms the measurement vector *z*.

(67) $$z=\left[\begin{array}{c} v_{N(GPS)}-v_{N(IMU)}\\ v_{E(GPS)}-v_{E(IMU)}\\ v_{D(GPS)}-v_{D(IMU)}\\ lat_{\left(GPS\right)}-lat_{\left(IMU\right)}\\ lon_{\left(GPS\right)}-lon_{\left(IMU\right)}\\ alt_{\left(GPS\right)}-alt_{\left(IMU\right)} \end{array}\right].$$

The latitude and longitude measurements are in radians with very small values, which can make the measurement matrix *H* singular. This can cause numerical instabilities in Kalman gain *K* calculation. So the measurement vector can be modified as:

(68) $$z=\left[\begin{array}{c} v_{N(GPS)}-v_{N(IMU)}\\ v_{E(GPS)}-v_{E(IMU)}\\ v_{D(GPS)}-v_{D(IMU)}\\ \left(lat_{\left(GPS\right)}-lat_{\left(IMU\right)}\right)\left(R_{E}+h\right)\\ \left(lon_{\left(GPS\right)}-lon_{\left(IMU\right)}\right)\left(R_{N}+h\right)\cos\lambda \\ alt_{\left(GPS\right)}-alt_{\left(IMU\right)} \end{array}\right].$$

The measurement matrix (H) is written as:

(69) $$H=\left\{\begin{array}{ccccccccc} 0 & 0 & 0 & 1 & 0 & 0 & 0 & 0 & 0\\ 0 & 0 & 0 & 0 & 1 & 0 & 0 & 0 & 0\\ 0 & 0 & 0 & 0 & 0 & 1 & 0 & 0 & 0\\ 0 & 0 & 0 & 0 & 0 & 0 & r_{N} & 0 & 0\\ 0 & 0 & 0 & 0 & 0 & 0 & 0 & r_{E}\cos L & 0\\ 0 & 0 & 0 & 0 & 0 & 0 & 0 & 0 & 1 \end{array}\right\}$$

All navigation calculations are being carried out in NED frame of reference.

## 5. Simulation, Data Collection and Results

The field test drive for data collection each lasting for fifteen minutes was carried out or Murree road (34.156024${}^{\circ }$ N, 73.222560${}^{\circ }$ E), Abbottabad city, Pakistan in January 2019. A total of twenty GPS and IMU measurement data sets were gathered over a period of three days. Two of the data sets were discarded due to erroneous data as a result of a hardware malfunction. Due to mountainous terrain of the city, satellite coverage was moderate and the average number of satellites available was equal to six. In order to avoid any timing errors between the GPS and IMU data, software written in MATLAB was used to time stamp the MEMS IMU data.

The YH-7000 commercial grade MEMS IMU was used for inertial measurements, which include angular rates and specific force data provided by three dimensional gyros and accelerometers. IMU data was obtained at 200 Hz. Simulations have been carried out on real vehicle test drive data in MATLAB R2014A platform (3.1 GHz, Intel, Core i5 CPU). In order to better visualize the filter’s performance for highly dynamic systems, Gaussian noise was introduced to the measurements during the interval between 300 s and 500 s. The actual GPS data was used to compare the estimation results. A total of 250 Monte Carlo simulations were carried out to omit any bias introduced by adding the noise.

**Figure 3:**
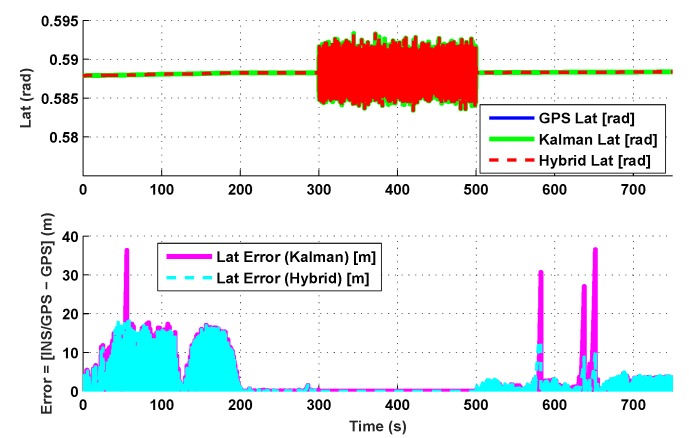
Latitude comparison.

**Figure 4:**
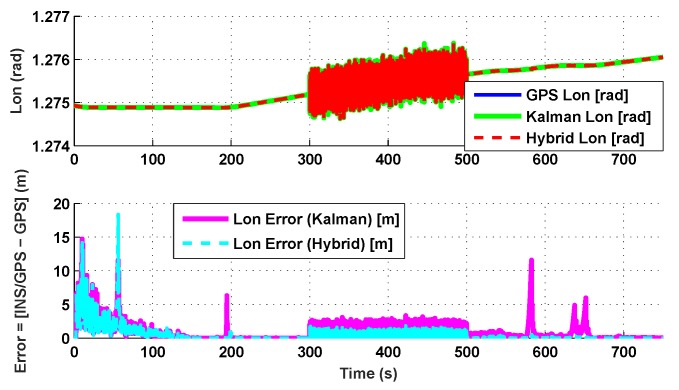
Longitude comparison.

**Figure 5:**
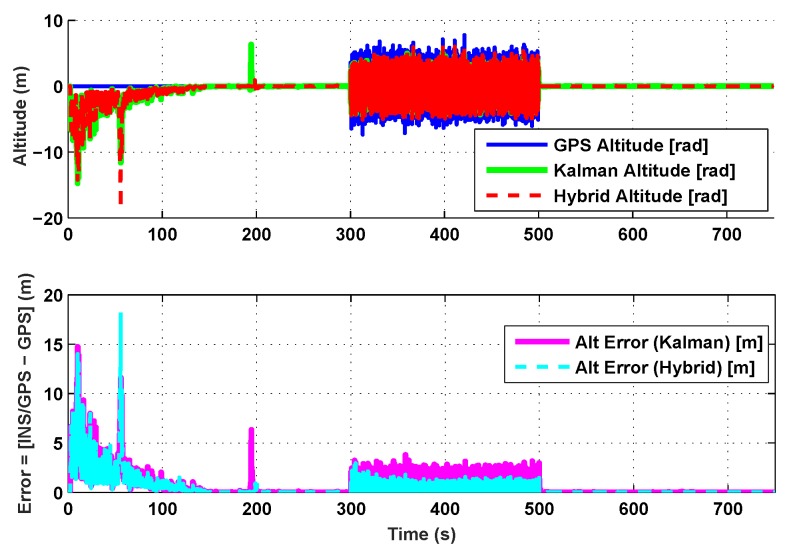
Altitude comparison.

**Figure 6:**
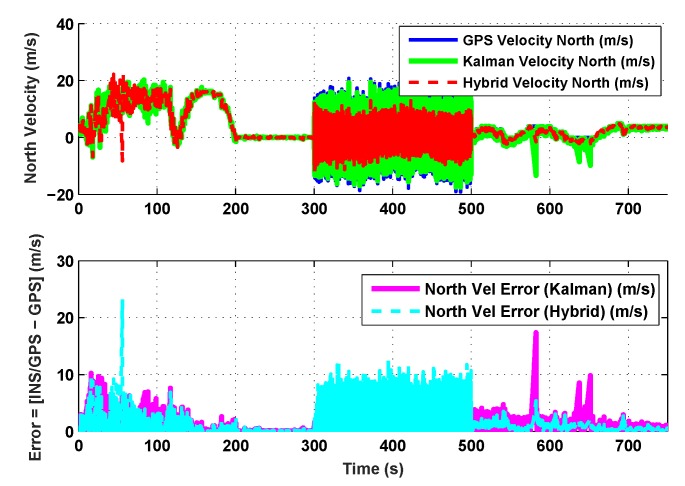
North velocity comparison.

**Figure 7:**
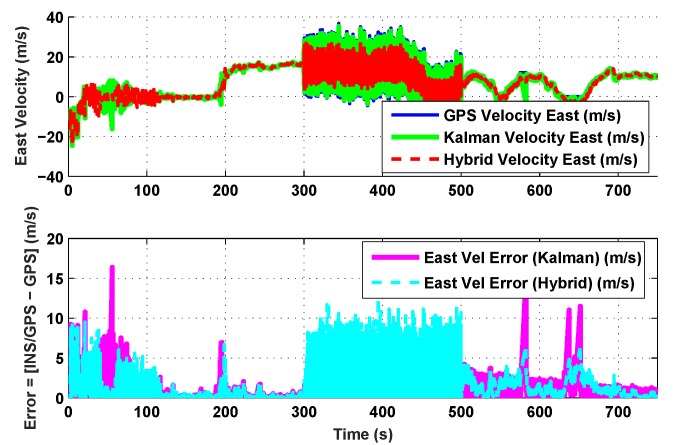
East velocity comparison.

**Figure 8:**
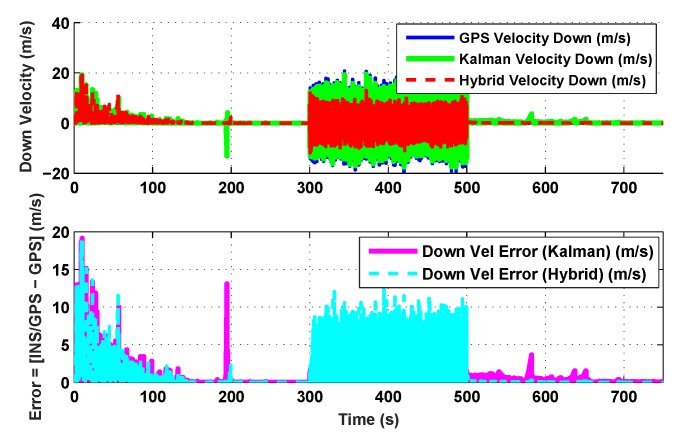
Down velocity comparison.

Novatel’s OEMV series GPS receiver was used to obtain raw data which was converted to position and velocity measurements using the on-board software at 1 Hz. The GPS measurements included vehicle’s position in terms of latitude, longitude, and altitude, and vehicle’s velocity in terms of north, east and down velocities. Typical error of commercial GPS data is about 30 m in latitude and longitude, while error in altitude is about 1.5 times greater than latitude/longitude error. The YH-7000 commercial grade MEMS IMU Sensor specifications are given in Table 1:

The results for the conventional linear Kalman filter and the hybrid adaptive Kalman filter for INS-GPS integrated navigation are now compared, as shown in the following simulation results with actual sensor data. Vehicle position in terms of latitude, longitude, and altitude is shown first in Figure 3, Figure 4 and Figure 5, whereas vehicle’s velocity in terms of north, east, and down components is shown in Figure 6, Figure 7 and Figure 8, respectively.

The proposed hybrid filter produces better estimates for vehicle’s position and velocity. Superior performance of the hybrid filter is more evident during the interval between 300 s and 500 s, when random noise was added to the actual sensor measurements. It converges more rapidly compared to the conventional Kalman filter. Similarly, when there is abrupt change in vehicle’s latitude and longitude measurements (near time = 650 s), the hybrid adaptive filter clearly excels at convergence (Figure 3 and Figure 4). This behavior is more pronounced for vehicle’s velocity estimation, which is understandable, given the fact that measurements have been taken for a ground vehicle, which generally experiences more abrupt changes in its velocity/acceleration rather than its position. Figure 6 and Figure 7 clearly show this trend for vehicle’s north and east velocity components.

**Figure 9:**
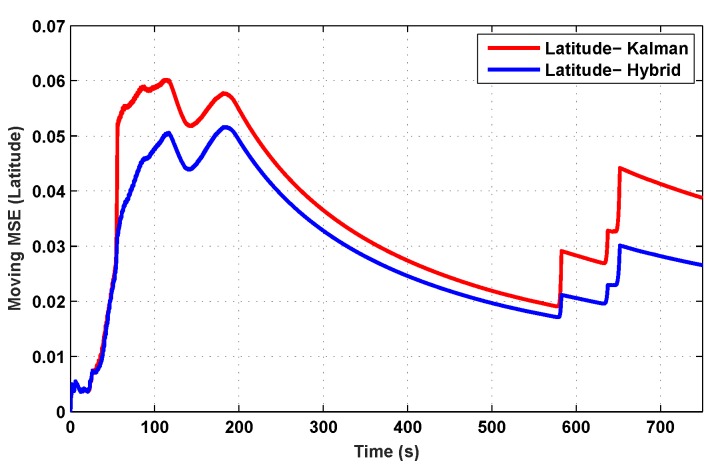
Moving mean square error (MMSE) comparison in latitude.

**Figure 10:**
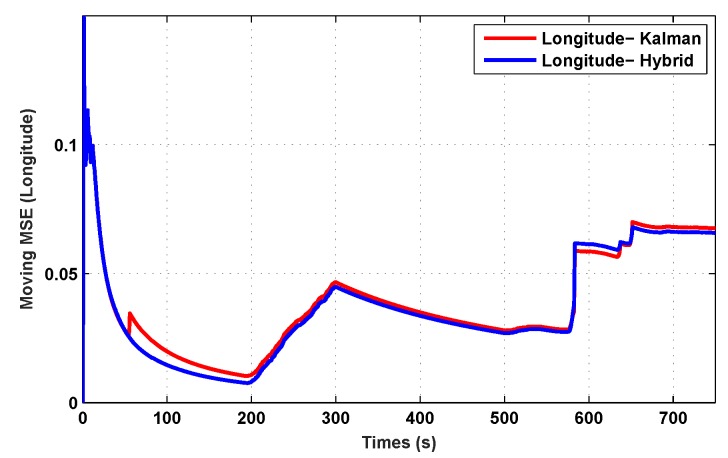
MMSE comparison in longitude.

**Figure 11:**
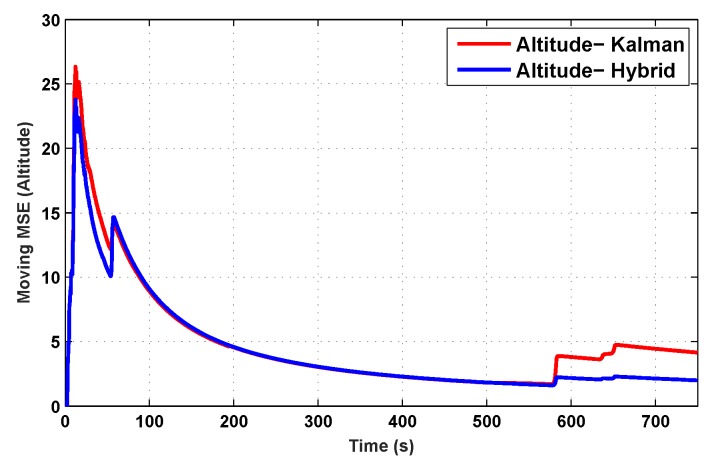
MMSE comparison in altitude.

**Figure 12:**
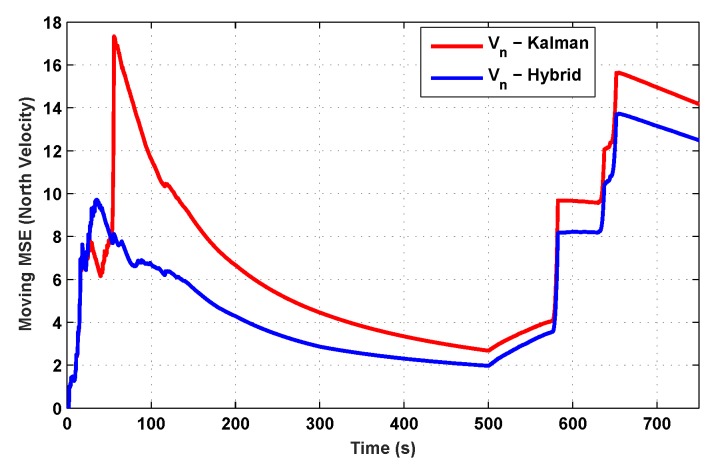
MMSE comparison in northward velocity.

**Figure 13:**
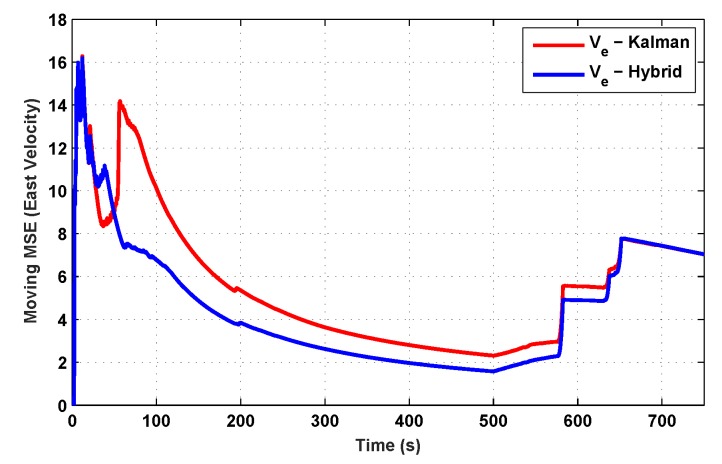
MMSE comparison in eastward velocity.

**Figure 14:**
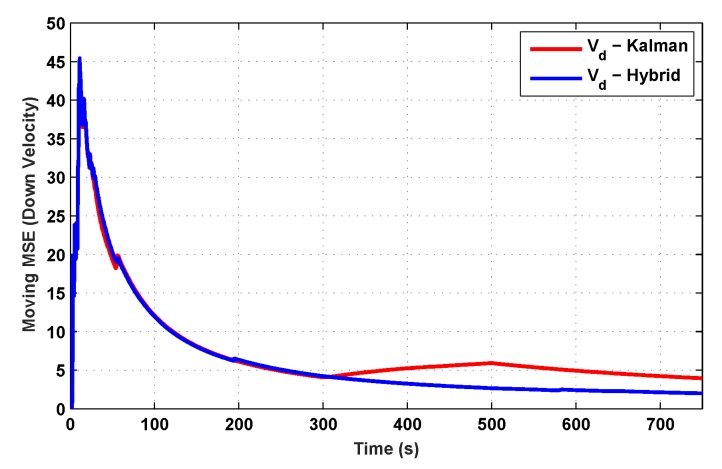
MMSE comparison in downward velocity.

**Figure 15:**
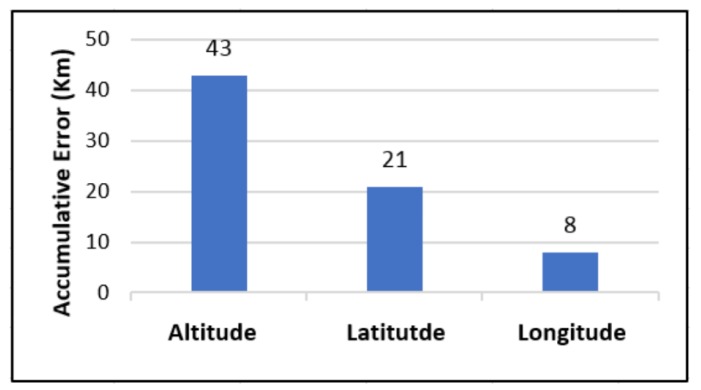
Difference between errors in position: proposed versus Kalman.

**Figure 16:**
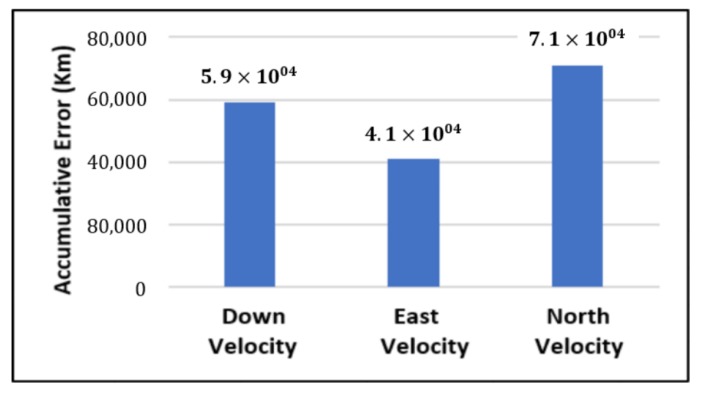
Difference between errors in velocity: proposed versus Kalman.

The position and velocity estimation curves for both the linear Kalman filter and the hybrid adaptive Kalman filter appear to be close and the two filters generate almost similar results. However, the hybrid filter, in effect, performs better, which can be seen from the moving mean square error (MMSE) plots of vehicle’s position and velocity (Figure 9, Figure 10, Figure 11, Figure 12, Figure 13, Figure 14 and Figure 15). Overall improvements made by the hybrid filter in vehicle’s position and velocity estimates are also shown by the accumulative error plots of vehicle’s position and velocity in Figure 15 and Figure 16.

The moving mean square error (MMSE) plots provided in Figure 9, Figure 10, Figure 11, Figure 12, Figure 13, Figure 14 and Figure 15 offer a better insight into the performance of proposed hybrid adaptive filter compared to conventional Kalman filter. These curves clearly illustrate superior estimation accuracy of the proposed filter scheme. A quantitative measure of the filter’s accuracy has also been provided in terms of accumulative error plots (Figure 15 and Figure 16), which show overall improvements made by the proposed hybrid scheme in vehicle’s position and velocity estimates.

These results prove the efficiency of the proposed scheme for parameter estimation in navigation, especially for the case when measurements contain large uncertainties/errors, or when states are changing more abruptly, such as the vehicle’s velocity.

Switching decision between the Sage-Husa filter and the robust tracking filter is made based on the check provided in Equation (45). When an anomaly in the measurements is detected, like a large error, or abrupt state change, the condition in Equation (45) is satisfied, and the filter switches to robust mode where more weight is assigned to latest measurements, thus, providing strong tracking of the target.

## 6. Conclusions

A novel algorithm based on a hybrid of the Sage-Husa filter and the strong tracking filter using a loosely coupled scheme is proposed for GPS-INS integration. The proposed algorithm has generated superior navigation for a vehicle’s position and velocity estimates. The algorithm offers a balance between accuracy and strong convergence for optimum estimation for highly dynamic systems. It inherently performs much better when implemented for more dynamic systems, as proven by the results. The same hybrid filter will, thus, produce even better navigation estimates for target tracking applications or in case of aerial vehicles, where a conventional Kalman filter will certainly lag behind due to its reliance on *a priori* information, and consequently, its inability to keep track of sudden changes in system states. The hybrid filter based on Sage-Husa–Kalman filter and strong tracking Kalman filter can, thus, be acknowledged as an optimal filter when compared to the conventional Kalman filter. 

## Figures and Tables

**Table 1:** IMU Sensor specifications.

Parameter	Gyro	Accelerometer
**Bias Repeatability**	<0.02${}^{\circ }$/s, 1 σ	<2 mg, 1 σ
**Random Walk**	<6${}^{\circ }$/hr${}^{1/2}$	<0.3 m/s2/hr${}^{1/2}$
**Scale Factor Stability**	<0.3%, 1 σ	<0.2%, 1 σ
**Bias Variation**	<0.1${}^{\circ }$/s, 1 σ	<5 mg, 1 σ
**Bandwidth**	>100 Hz, Gain @ −3 dB	>100 Hz, Gain @ −3 dB
